# *Escherichia coli* as Commensal and Pathogenic Bacteria among Food-Producing Animals: Health Implications of Extended Spectrum β-Lactamase (ESBL) Production

**DOI:** 10.3390/ani10122239

**Published:** 2020-11-29

**Authors:** Sónia Ramos, Vanessa Silva, Maria de Lurdes Enes Dapkevicius, Manuela Caniça, María Teresa Tejedor-Junco, Gilberto Igrejas, Patrícia Poeta

**Affiliations:** 1 Microbiology and Antibiotic Resistance Team (MicroART), Department of Veterinary Sciences, University of Trás-os-Montes and Alto Douro (UTAD), 5001-801 Vila Real, Portugal; soniacatarinaramos@gmail.com (S.R.); vanessasilva@utad.pt (V.S.); 2 Department of Genetics and Biotechnology, University of Trás-os-Montes and Alto Douro, 5001-801 Vila Real, Portugal; gigrejas@utad.pt; 3 Functional Genomics and Proteomics Unit, University of Trás-os-Montes and Alto Douro (UTAD), 5001-801 Vila Real, Portugal; 4 Associated Laboratory for Green Chemistry (LAQV-REQUIMTE), University NOVA of Lisboa, 2829-516 Lisbon, Portugal; 5 Faculty of Agricultural and Environmental Sciences, University of the Azores, 9500-321 Angra do Heroísmo, Portugal; maria.ln.dapkevicius@uac.pt; 6 Institute of Agricultural and Environmental Research and Technology (IITAA), University of the Azores, 9500-321 Angra do Heroísmo, Portugal; 7 National Reference Laboratory of Antibiotic Resistances and Healthcare Associated Infections (NRL-AMR/HAI), Department of Infectious Diseases, National Institute of Health Dr Ricardo Jorge, Av. Padre Cruz, 1649-016 Lisbon, Portugal; Manuela.Canica@insa.min-saude.pt; 8 Centre for the Studies of Animal Science, Institute of Agrarian and Agri-Food Sciences and Technologies, Oporto University, 4051-401 Oporto, Portugal; 9 Research Institute of Biomedical and Health Sciences, University of Las Palmas de Gran Canaria, 35001 Canary Islands, Spain; mariateresa.tejedor@ulpgc.es; 10 Department of Clinical Sciences, University of Las Palmas de Gran Canaria, 35001 Canary Islands, Spain

**Keywords:** *Escherichia coli*, ESBL, food-producing animals, antimicrobial resistance

## Abstract

**Simple Summary:**

This revision is about the problem of *Escherichia coli* as a commensal and pathogenic bacterium among food-producing animals and health implications. Escherichia coli may play an important ecological role and can be used as a bioindicator of antimicrobial resistance. All animal species used for food production, as well as humans, carry *E. coli* in their intestinal tract; plus, the genetic flexibility and adaptability of this bacteria to constantly changing environments allows it to acquire a great number of antimicrobial resistance mechanisms. The majority of *E. coli* strains are commensals inhabiting the intestinal tract of humans and warm-blooded animals and rarely causes diseases. However, *E. coli* also remains as one of the most frequent causes of several common bacterial infections in humans and animals. All over the word, antibiotic resistance is commonly detected among commensal bacteria from food-producing animals, raising important questions on the potential impact of antibiotic use in animals and the possible transmission of these resistant bacteria to humans through the food chain. The use, in food-producing animals, of antibiotics that are critically important in human medicine has been implicated in the emergence of new forms of resistant bacteria, including new strains of multidrug-resistant foodborne bacteria, such as extended spectrum β-lactamase (ESBL)-producing *E. coli*.

**Abstract:**

*Escherichia coli* are facultative, anaerobic Gram-negative rods with many facets. Within resistant bacterial populations, they play an important ecological role and can be used as a bioindicator of antimicrobial resistance. All animal species used for food production, as well as humans, carry *E. coli* in their intestinal tracts; plus, the genetic flexibility and adaptability of this bacteria to constantly changing environments allows it to acquire a great number of antimicrobial resistance mechanisms. Thus, the prevalence of antimicrobial resistance in these commensal bacteria (or others, such as enterococci) can be a good indicator for the selective pressure caused by the use of antimicrobial agents, providing an early warning of the emergence of antimicrobial resistance in pathogens. As many as 90% of *E. coli* strains are commensals inhabiting the intestinal tracts of humans and warm-blooded animals. As a commensal, it lives in a mutually beneficial association with its hosts and rarely causes diseases. However, *E. coli* also remains as one of the most frequent causes of several common bacterial infections in humans and animals. In humans, it is the prominent cause of enteritis, community- and hospital-acquired urinary tract infection (UTI), septicemia, postsurgical peritonitis, and other clinical infections, such as neonatal meningitis, while, in farm animals, it is more prominently associated with diarrhea. On a global scale, *E. coli* can be considered the most important human pathogen, causing severe infection along with other major bacterial foodborne agents, such as *Salmonella* spp. and *Campylobacter*. Thus, the importance of resistance in *E. coli*, typically considered a benign commensal, should not be underestimated.

## 1. Pathogenicity of *Escherichia coli*

*E. coli* is a ubiquitous commensal of food-producing animals and humans. Most strains of this enterobacterial species are harmless commensals that live in a mutually beneficial association with their hosts and seldom cause disease. *E. coli* is, however, a particularly complex species, having diversified into pathogenic strains. Based on the type of virulence factor present, and the host’s clinical symptoms, *E. coli* strains are classified into pathotypes of zoonotic intestinal pathogenic *E. coli* (IPEC) or extraintestinal pathogenic *E. coli* (ExPEC) [1].

Within the IPEC, the diarrheagenic *E. coli* (DEC) groups include enteropathogenic *E. coli* (EPEC), enterotoxigenic *E. coli* (ETEC), enteroinvasive *E. coli* (EIEC), enteroaggregative *E. coli* (EAggEC), diffusely adherent *E. coli* (DAEC), enterohemorrhagic *E. coli* (EHEC) and Vero cytotoxin-producing *E. coli* (VTEC) or Shiga toxin-producing *E. coli* (STEC) [2]. Food poisoning outbreaks have been particularly associated with VTEC and, to a lesser extent, EPEC, ETEC and EAggEC strains [3]. The *E. coli* O157:H7 VTEC strain has become widely recognized as a very important cause of foodborne illness [4]. Since 1982, outbreaks have been recorded in the U.S. and throughout Europe. The source most often found to be contaminated was beef meat, often minced, but today, the organism is widespread in the guts of asymptomatic cattle, and their feces can potentially contaminate other products (like vegetables, sprouts, fruits, meat products, drinking water, juices and milk) [5,6].

The ExPEC group brings together the uropathogenic *E. coli* (UPEC), the neonatal meningitis *E. coli* (NMEC) and the avian pathogenic *E. coli* (APEC), which are frequently associated with nosocomial and community-associated infections [7]. Poultry meat is the food of the animal source most closely linked to human ExPEC. In addition to the overall highest levels of *E. coli* contamination found in poultry meat, virulence genes similar to those of human ExPEC are often found in poultry-associated *E. coli* strains [8]. Moreover, extensive genetic similarity has been documented between APEC and ExPEC strains, causing disease in poultry and humans, respectively [8,9]. Although beef and pork meats were also evaluated as potential reservoirs of ExPEC causing urinary tract infections (UTIs) in humans, the recovered ExPEC isolates were significantly less likely to be genetically related to isolates from humans with UTIs than those from poultry [10]. Some of the emerging ExPEC lineages associated with human outbreaks are known to be linked with food-producing animals. For example, *E. coli* O25:H4-B2-ST131 has a globally emerging lineage with an extensive antimicrobial resistance profile, including the CTX-M-15 enzyme, and fluoroquinolone resistance [10]. In addition, wild, companion and food-producing animals have been reported as carriers of this group [11]. Furthermore, *E. coli* (various serotypes)-A-ST10, a commonly encountered, antimicrobial susceptible, low-virulence, human intestinal colonizer has been associated with some human infections and extended spectrum β-lactamase (ESBL) production [11,12]. Moreover, the ESBL-producing *E. coli* ST10 has been recovered from chicken meat, other meat types, rectal swab samples from healthy humans and human blood cultures [13,14].

The presence of several putative virulence genes enables pathogenic ExPEC bacteria to cause infections. According to their phylogenetic classification, ExPECs typically belong to group B2 and, less commonly, to group D, whereas commensal intestinal strains belong to group A or B1 [15]. However, virulence-associated genes, in and of themselves, rarely make an organism virulent. Their levels of expression, which can vary between pathogenic and nonpathogenic isolates, can also be a determining factor [16]. Moreover, it seems that these putative virulence factors, rather than being directly involved in infection, also contribute to ExPEC fitness, increasing their adaptability, competitiveness and ability to colonize the human body [16]. ExPEC strains are characterized by virulence factors that may be present in various combinations, including adhesins (*papC*, F10*pap*A, *sfa*DE, *afa*BC III, *iha*, *fimH*, *clp*G, *tsh* and *hra*); invasin (*ibe*10); iron-sequestering systems (*iucD*, *irp*2 and *chuA*); toxins (*omp*T, *ehxA*, *espP*, *hlyA*, *hlyD*, *vat*, *sat* and *cnf*1); capsules (K1, K5, *kps*MT II and *kps*MT III); siderophores (*iro*N, *fyu*A and *ire*A) and various other factors (*iss*, *usp*, *tra*T, *mal*X, *cva*C and H7 *fli*C) [17].

## 2. Antimicrobial Resistance Trends in *E. coli*

The presence of mobile genetic elements such as plasmids, insertion sequences and transposons contributes to the plasticity of *E. coli*’s genome. Horizontal gene transfer has promoted the diffusion of antibiotic resistance genes among this species and other commensals [18], particularly in environments such as the intestinal tract, where the species diversity and bacterial population density are large. Therefore, *E. coli* has been used as a sentinel microorganism for antimicrobial resistance surveillance, especially in the case of the β-lactams [19].

Antimicrobial-resistant *E. coli* strains are broadly distributed in Europe, both in humans and in food-producing animals. As reported in the 2018 Annual Report of the European Antimicrobial Resistance Surveillance Network (EARS-Net), more than half of the *E. coli* isolates in Europe were resistant to at least one class of antimicrobials. Aminopenicillin resistance, followed by a resistance to fluoroquinolones, third-generation cephalosporins and aminoglycosides, were the most prevalent [20]. Furthermore, the reduction of antimicrobial-resistant *E. coli* in Europe was very low or nonexistent between 2015 and 2018, with *E. coli* being the major burden of antimicrobial resistance both in the number of cases and number of deaths [20]. Meanwhile, among food-producing animals, the European Food Safety Authority (EFSA) reported that the high proportions of *Salmonella*, *Campylobacter* and indicator *E. coli* isolates exhibiting reduced susceptibility to fluoroquinolones remain of concern [21]. However, co-resistance to “clinically important antimicrobials”, such as a resistance to third-generation cephalosporins or fluoroquinolones, are generally reported at very low to low levels in commensal *E. coli* isolates from animals [21].

These trends among food-producing animals are also evidenced by several other reports. Resistance to third-generation cephalosporins and quinolones was found among clinical *E. coli*, already resistant to most antimicrobials available for poultry, in a study conducted in 200 industrial poultry farms in Italy [22]. Likewise, in Germany, animals from 60 beef cattle and 52 dairy cattle production units were sampled; third-generation cephalosporin-resistant *E. coli* were isolated from at least one sample in 70% of the beef cattle farms and 85% of the dairy farms [23]. Besides resistances to antimicrobial classes that have been extensively used for a long time (e.g., sulphonamides and tetracyclines), high resistance rates to ciprofloxacin were also found among isolates from food-producing animals, more often in broilers, chicken meat and turkey meat than in the cattle and pig production chains [24,25,26]. These reports notwithstanding, *E. coli* with resistance to “critically important” antibiotics (especially to quinolones but, also, to colistin) in food-producing animals has been increasingly reported by others, as well as multidrug resistance (MDR) in commensal *E. coli* [27,28,29,30,31,32,33,34]. Moreover, the worldwide dissemination of MDR *E. coli* strains is mainly due to the spread of genes located on mobilizable genetic elements, including integrons, plasmids and transposons [35,36,37,38,39]. Since quinolones, colistin and third-generation cephalosporins are priority antimicrobials in human antimicrobial therapy, the emergence of this resistance warrants special concern and requires close monitoring.

## 3. The Major Threat from ESBL-Producing *E. coli*

### 3.1. Contextualizing the Issue

ESBL production is of importance not only in the nosocomial and long-term care perspectives but, also, for community-onset infections, with increasing reports of the latter. The transmission of ESBL-producing bacteria may occur from human to human, or from animal sources to humans via the food chain [40]. The clinical impact of infections by ESBL-producing *E. coli* strains has mainly been studied in hospitalized patients. These infections present a higher mortality rate associated with a delay in implementing an appropriate antimicrobial therapy, since empirically prescribed antibiotics may not be effective in this case [40]. In a study in Europe [41], ESBL-producing *E. coli* infections were estimated to be in the order of 300,000 and to have caused 9000 attributable deaths. Besides, infections with ESBL-producing bacteria have been associated with longer hospital stays and with an increased burden on the health services [42]. An overall global burden of the infections by ESBL-producing *E. coli* in farm animals is yet to be estimated, but their potential role as a reservoir of these microorganisms for humans necessitates a One Health, interdisciplinary approach to keep this threat under control [43].

Organisms producing ESBLs have been increasingly reported worldwide since their first description in Europe in the early 1980s [44]. A recent study reported a cumulative global pooled prevalence of ESBL-producing *E. coli* intestinal carriage, in the community, of 16.5% for the 2013–2018 period, with an eight-fold increase during the past two decades, indicating that preventing the dissemination of such strains may require new therapeutic and public health strategies [45].

The presence of ESBLs and their combined resistance is a serious public health concern. In addition to the resistance conferred by the ESBL enzymes, co-resistance to other antibiotic classes is frequently observed, drastically limiting therapeutic options available and putting human health in peril [46]. Moreover, this phenomenon may lead to the increased use of carbapenems, favoring further dissemination of carbapenemase-producing *Enterobacteriaceae* [47].

ESBLs are, mostly, plasmid-encoded enzymes that confer extended resistance to β-lactams (penicillins, first-, second- and third-generation cephalosporins and aztreonam) but not to cephamycins or carbapenems [48]. They are inhibited by β-lactamase inhibitors such as clavulanic acid and belong to Ambler’s classes A and D [49]. ESBLs are found in Bush-Jacoby-Medeiros classes 2be and 2d [50]. ESBL production is common in *E. coli* and *Klebsiella pneumoniae*, but it also occurs in other Enterobacteriaceae and in *Pseudomonas aeruginosa.* ESBL have been classified in three major subtypes: TEM, SHV and CTX-M β-lactamases. ESBLs derived from Bush-Jacoby-Medeiros type 2b enzymes—the TEM- and SHV-type ESBLs—are a large, widespread group and differ from their parental enzymes by as few as one or two amino acids [51]. These minor differences in their amino acid sequences are, nevertheless, enough to extend the spectrum of their enzymatic activity, making them able to hydrolyze cephalosporins that have an oxyimino side chain (third-generation cephalosporins and aztreonam) [50]. Contrarily to other ESBLs, the CTX-M family is a heterogenous, complex group of enzymes that possibly derived from the relocation of chromosomal *Kluyvera* genes to mobile genetic elements, conferring resistance to cefotaxime and ceftazidime [52].

For a long time, TEM and SHV types were the dominant ESBL enzymes all over the world. However, this situation changed dramatically in the present century. Nowadays, CTX-M enzymes have become the most widespread type of ESBLs [53]. Worryingly, most ESBL-producing isolates are now *E. coli*-expressing CTX-M β-lactamases that, in the modern landscape, “crossed the border” from hospital settings to the community [42]. We are now witnessing a worldwide epidemic of *E. coli* strains harboring CTX-M enzymes that require serious attention. The CTX-M-15 enzymes are, by far, the most globally spread—virtually present in all human and animal compartments, as well as in the environment—all over the world [54,55,56,57].

### 3.2. CTX-M β-Lactamases and Their Relevance

The CTX-M enzymes constitute a distinct phylogenetic lineage of molecular class A β-lactamases. The first *bla*_CTX-M_ was described in 1990, from a clinical *E. coli* isolate, in Germany [58]. Nowadays, at least 170 members of the CTX-M family have been identified, in at least 26 bacterial species, but the majority are from *E. coli*, *K. pneumoniae* and *Proteus mirabili*s [59]. Based upon their amino acid homology, the CTX-Ms can be divided into five main groups: Group 1 (CTX-M-1, 3, 10, 11, 12, 15, 22, 23, 28, 29, 30 and UOE- 1), Group 2 (CTX-M-2, 4, 5, 6, 7 and 20), Group 3 (CTX-M-8), Group 4 (CTX-M-9, 13, 14, 16, 17, 19, 21 and 27) and Group 5 (CTX-M-25 and 26) [60].

The CTX-M genes can be traced back to the chromosome-encoded genes of *Kluyvera* spp., strongly indicating that these chromosomal β-lactamase genes were the probable progenitors of all groups of plasmid-mediated CTXMs [58]. The original *Kluyvera bla*_CTX-M_ genes conferred hydrolytic activity against cefotaxime to a higher degree than against ceftazidime. Later on, similarly to other ESBLs, divergence by point mutations led to increased activity against the latter antimicrobial as well [61].

### 3.3. Dissemination of CTX-M and Its Implications

In *Enterobacteriaceae*, the great adaptive success of *bla*_CTX-M_ genes has been associated with a few surrounding genetic structures and a few plasmids [62,63]. The spread of CTX-M is dependent upon its mobility. The mobilization of *bla*_CTX-M_ genes from their original chromosomal position in *Kluyvera* species has been facilitated by mobile genetic elements, such as ISE*cp1* or IS*CR1*, and their later incorporation in mobilizable genetic platforms, including class 1 integrons, plasmids and/or transposons [64]. The insertion sequence ISE*cp1* provides a high-level expression promoter for the *bla*_CTX-M_ gene and is positioned upstream of *bla*_CTX-M_ genes from group CTX-M-1, -2, -9 and -25 enzymes [58,65]. Furthermore, the ISE*cp*1 element has been linked to other multiple resistance determinants (*bla*_CMY_, *aph*(2′), *rmt*C and *qnr*) in various members of the *Enterobacteriaceae* family, underlining their ability to facilitate the expression and spread of different antimicrobial resistance determinants [58]. More intriguingly, the IS*CR* can mobilize the *bla*_CTX-M_ gene via rolling circle transposition and insertion into a class 1 integron, providing a putative promoter for high-level expression [58,66].

It is acknowledged that, once transferred on plasmid(s) and/or integron(s), the *bla*_CTX-M_ genes have broader opportunities for horizontal spread among different Gram-negative organisms, and these mobile elements became one important factor in the epidemiology of this bacterial ecosystem [67]. Epidemiological studies based on molecular techniques have revealed a close and significant linkage of *bla*_CTX-M_ genes to plasmids, mainly of the IncF, IncI, IncN, IncHI2, IncL/M and IncK groups [65,68]. The gene *bla*_CTX-M-15_ has been often identified in IncF group plasmids (FIA, FIB and FII), while IncF, IncK and IncI1 are closely related to the wide spread of *bla*_CTX-M-14_ genes [58,65]. In addition, CTX-Ms, such as *bla*_CTX-M-1_ and *bla*_CTX-M-3_, have frequently been reported on broad host-range replicon plasmids IncN, IncI1 and IncL/M [62]. Moreover, the presence of IncN plasmids carrying *bla*_CTX-M-1_ in farm animals, as well as their spread between humans and animals, have been documented, with the IncN and IncI1 plasmids found to be highly prevalent among *E. coli* from pig fecal microbiota [69]. Furthermore, genes encoding a resistance to quinolones, aminoglycosides, macrolides, tetracyclines, sulfonamides, trimethoprim and chloramphenicol have all been associated with *bla*_CTX-M_-containing plasmids [58]. The successful associations of these units, and the coexistence of *bla*_CTX-M_ genes with other resistance determinants, might have contributed to the extraordinary spread of CTX-M enzymes and to the present uncontrolled pandemic scenario. On the other hand, the coexistence of two or more β-lactamases in the same strain is frequently found [52]. Moreover, there is little doubt that the use of cephalosporins and related compounds has been one of the driving forces in the persistence and dissemination of ESBL-producing bacteria, with the use of other antimicrobial compounds also exerting the same selective force [68].

One of the most interesting issues in the dispersion of CTX-M enzymes is the dissemination of specific clones. The application of Multi-Locus Sequence Typing (MLST) technologies to the study of CTX-M-15-producing ESBL isolates led to the recognition of the internationally disseminated clone B2,O25:H4-ST131 [66,70]. There is great concern about the pandemic spread of the CTX-M-15-producing *E. coli* of Sequence Type (ST) 131 [66]. This clone belongs to the phylogenetic group B2, a highly virulent group of ExPEC, which is responsible for urinary tract infections, bacteremia, urinary sepsis and neonatal sepsis [71,72]. Further, ST131 is the most studied phylogenetic lineage in terms of antimicrobial resistance in *E. coli*, and *bla*_CTX-M-15_-carrying strains of ST131 are often associated with other resistance determinants, such as trimethoprim-sulfamethoxazole, aminoglycosides, fosfomycin and fluoroquinolones [58]. In addition, they have been associated with 100% of ciprofloxacin resistance rates, close to those of the aminoglycosides [73]. The combination of virulence and antimicrobial resistance may give *E. coli* ST131 a fitness advantage over other *E. coli* strains. Furthermore, the spread of ST131 occurred among human isolates, but it is also disseminated to various animal species, including poultry, cattle, pigs, wildlife and pets [71]. Other *E. coli* lineages of the “virulent” phylogroup D, also associated with multi-resistance, include ST69, ST405 and O15:K52:H1 [11,74]. In addition, *E. coli* clones belonging STs 10 and 23, and to phylogroup A, are increasingly reported in association with ESBL production [75].

**Figure 1:**
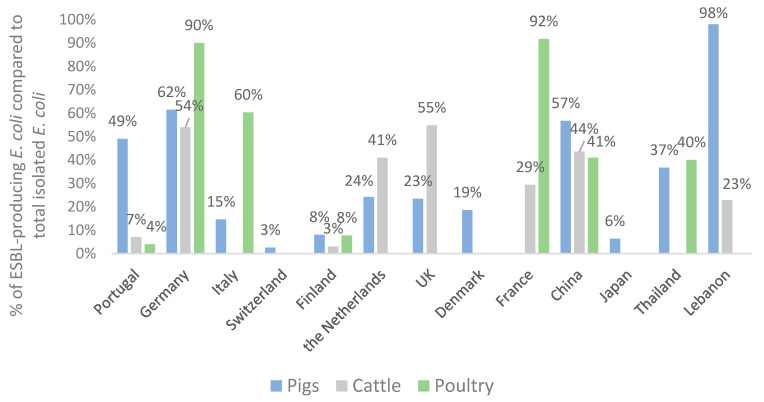
Percentage of extended spectrum β-lactamase (ESBL)-producing *Escherichia coli* strains reported in healthy food-producing animals in different countries [23,77,78,79,80,81,82,83,84,85,86,87,88,89,90,91,92,93,94,95,96,97,98].

It is worrisome that commensal *E. coli* bacteria from food-producing animals may contribute to the dissemination of ESBL resistance and to the transference of resistance genes to human pathogenic bacteria, such as *Salmonella* spp. [76]. Figure 1 compares the percentages of ESBL-producing *E. coli* strains reported in food-producing animals by different European and non-European countries. For comparison purposes, we selected studies where samples were collected from healthy animals at the farm or slaughter level. Apart from Lebanon, Germany, China, Portugal and Thailand, where high percentages (>25%) of ESBL-producing *E. coli* were found among pig isolates, only low-to-moderate percentages (>1% to <25%) were detected in the other countries. Concerning cattle isolates, the overall results pointed towards lower percentages than in swine, but percentages were still high (>25%) in Germany, the Netherlands, the UK and China. Regarding the presence of ESBL-producing *E. coli* isolated from poultry, there was a very high percentage in Germany, France and Italy.

The variability across data can be explained by differences in husbandry practices, antibiotic usage or, even, in experimental methodologies. However, in most reports, the data from antibiotic use were not included, making it impossible to draw practical conclusions concerning this aspect. Furthermore, isolation methods (e.g., the use of different enrichment broths and/or selective media) and sample sizes vary greatly between countries, which may have resulted in different screening sensitivities. Hence, comparing prevalence data should, in some instances, be performed cautiously; for example, in the recent report by the EFSA, the overall levels of resistance to cefotaxime and ceftazidime in the reporting countries were generally low (<4%), both in pigs and in cattle [21]. It is of note that differences in the type of cattle (dairy cattle, veal calves or beef cattle) sampled should also be taken in consideration when comparing data. Therefore, the higher percentages of ESBL-producing *E. coli* obtained among cattle isolates in Germany can be explained by the type of cattle included in that study, in which samples were collected from mixed farms and beef cattle farms [23]. This fact can also be explained by farming differences, like housing and exposure to antibiotics. Conversely, in France, the study by Haenni et al. (2014), in which 29% of ESBL-producing *E. coli* were found, was performed at the slaughterhouse [83].

The globalization of CTX-M enzymes is illustrated by their presence not only in humans but, also, in food, food-producing, companion and wild animals, as well as in the environment [71]. Nowadays, the prevalence of food-producing animals carrying *E. coli* producing CTX-M-type ESBLs has reached worryingly high levels, raising questions about the possible role of animals and food, as related reservoirs, in this phenomenon. The high proportions of resistance to third-generation cephalosporins and fluoroquinolones reported for *E. coli* in humans are concerning; meaning that, in many settings, the treatment must rely on carbapenems, a more expensive drug, that may not be available in resource-constrained settings and which is also likely to further accelerate the development of resistance [99]. The rapid, global dissemination of the plasmid-borne, colistin resistance-encoding *mcr*-1 gene, since its discovery in 2016 and its presence in environmental sources, in animals and in food [100], as well as its co-occurrence with genes that encode ESBLs [92] and New Delhi Metallo-β-lactamase (NDM-1) [101], has also raised concerns about colistin resistance in *E. coli*.

### 3.4. Distribution of ESBL Enzymes in E. coli Isolates from Food-Producing Animals

**Figure 2:**
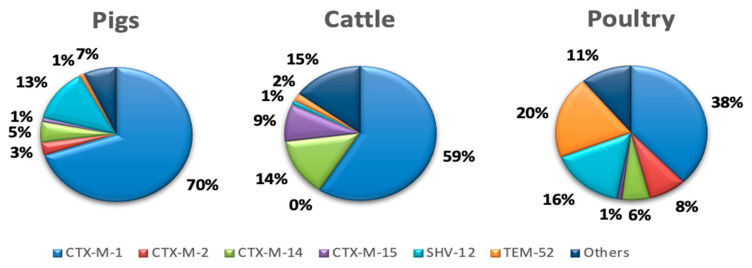
Distribution of ESBL enzymes in isolates from food-producing animals in Europe [102].

**Figure 3:**
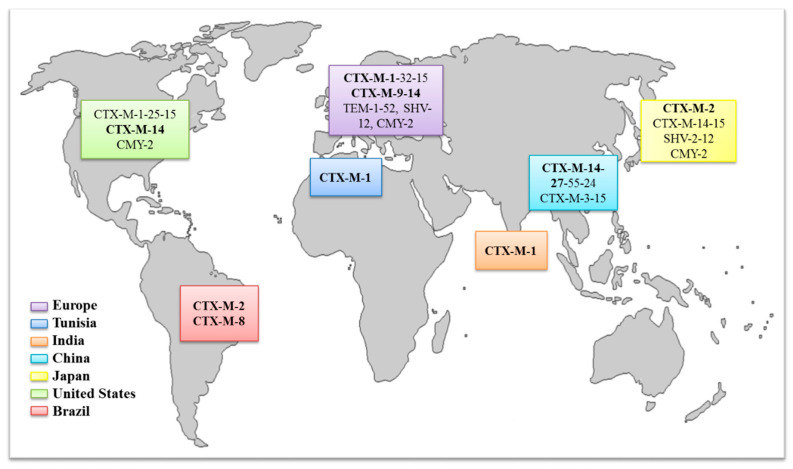
Global distribution of ESBL enzymes frequently (bold) detected in *E. coli* isolates from food-producing animals [71,103,104,105].

There are several different types of β-lactamase that can confer resistance to third-generation cephalosporins. These are conveniently subdivided into four classes, designated A to D; ESBL enzymes of the TEM, SHV and CTX-M families belong to class A, while class C includes the AmpC β-lactamases [52]. In animals, the most common genes associated with this resistance are *bla*_CTX-M-1_, *bla*_CTX-M-2_, *bla*_CTX-M-14_, *bla*_CTX-M-15_*, bla*_TEM-52_ and *bla*_SHV-12_ [102]. Figure 2 and Figure 3 show the distribution of ESBL enzymes detected in pigs, cattle and poultry in Europe and the global distribution of the ESBL enzymes most frequently detected in *E. coli* isolates from food-producing animals, respectively.

Looking at the world distribution of genes responsible for resistance to extended-spectrum cephalosporins, the CTX-M-1 group (CTX-M-1 and -15) is predominant in European countries. Additionally, the CTX-M-9 group (CTX-M-9 and -14) has been frequently identified in animals from Spain, Portugal and the United Kingdom. The CTX-M-2 group has been mainly described in South America and Japan, whereas, in China, enzymes of the CTX-M-9 group are prevalent, and in the United States or North Africa, the enzymes of the CTX-M-1 group are frequently identified. Apart from this general overview, CTX-M-15 and CTX-M-14 are, by far, the most important ones, since they are broadly distributed in food-producing animals and humans and are commonly detected in clinically important pathogens [105].

Nowadays, the global spread, and high prevalence, of CTX-M enzymes in *E. coli* is a matter of concern in both human and veterinary medicines. Although the dominant variants of CTX-Ms can be geographically different, in humans, CTX-M-15 and CTX-M-14 are among the most common variants detected worldwide in clinically important pathogens [71,105], whereas, overall, in food-producing animals, the most commonly reported genes encode for CTXM-type enzymes (e.g., CTX-M-1, -2, -9, -14, -15, -32 and -55), followed by SHV-12 and TEM-52 ESBLs [71,102]. In particular, the CTX-M-1 is broadly disseminated among food-producing animals in Europe. However, it is rarely reported in other regions and settings [102]. On the other hand, although CTX-M-15 has spread in a pandemic fashion in humans, they were only detected incidentally in poultry in European countries, whereas pets (15%) and cattle/pigs (8%) are frequently associated with this enzyme type [71]. Furthermore, the zoonotic potential of ESBL-producing bacteria and the fact that CTX-M expression is often associated with a co-resistance to other “critical important antibiotics” can have direct implications in human health by reducing treatment options. This underlines the need to consider carefully the use of fluoroquinolones, colistin or third- and fourth-generation cephalosporins as treatment options in animals in view of their critical importance in the treatment of systemic or invasive Gram-negative bacterial infections in humans.

## 4. Conclusions

All over the world, antibiotic resistance is commonly detected among commensal bacteria from food-producing animals, raising important questions on the potential impact of antibiotic use in animals and the possible transmission of these resistant bacteria to humans through the food chain. The use, in food-producing animals, of antibiotics that are critically important in human medicine has been implicated in the emergence of new forms of resistant bacteria, including new strains of multidrug-resistant foodborne bacteria, such as ESBL-producing *E. coli*. The ample, rapid dissemination of ESBLs among commensal and pathogenic *E. coli* strains, in humans, domestic animals and environmental sources, during the last two decades makes them an important threat in terms of public health. Addressing this challenge requires a continued research effort towards a better understanding of the nature and dissemination pathways of ESBLs and of the bacterial strains that bear them. A relentless vigilance of the evolution of the ESBL situation and the application of a One Health interdisciplinary approach is necessary to keep this problem under control.
